# Dislocation Dynamics-Based Modeling and Simulations of Subsurface Damages Microstructure of Orthogonal Cutting of Titanium Alloy

**DOI:** 10.3390/mi8100309

**Published:** 2017-10-16

**Authors:** Jinxuan Bai, Qingshun Bai, Zhen Tong

**Affiliations:** 1 School of Mechanical and Electrical Engineering, Harbin Institute of Technology, Harbin 150001, China; 2 Centre for Precision Technologies, University of Huddersfield, Huddersfield HD1 3DH, UK; Z.Tong@hud.ac.uk

**Keywords:** micro-cutting, dislocation dynamics (DD), subsurface damages microstructure, tool structure

## Abstract

In this work, a novel method is put forward to quantitatively simulate the subsurface damages microstructural alteration of titanium alloy components subjected to microscale cutting. A trans-scale numerical framework is conducted with the purpose of revealing the underlying influence mechanism of tool structure parameters on subsurface dislocation configurations using a dislocation dynamics-based model, which considers both dislocation structural transformation and grain refining. Results showed that the developed framework not only captured the essential features of workpiece microstructure, but also predicted the subsurface damages layer states and their modifications. A series of defects were found in the material subsurface during the orthogonal cutting of titanium alloy, such as edge and screw dislocations, junctions, parallel slip lines, intersection dislocation bands, vacancy defects, and refinement grains. Particularly, in the process of micro-cutting, the depth of subsurface damages layer increased significantly with cutting length at the beginning, and then remained unchanged in the stable removal phase. Moreover, smaller edge radius and larger rake angle can greatly weaken the squeezing action and heat diffusion effect between the tool tip and workpiece, which further prevents the formation of subsurface defects and enhances finished surface quality. In addition, although increasing tool clearance angle could drastically lighten the thickness of subsurface damages layer, it is noteworthy that its performance would be decreased significantly when the clearance angle was greater than or equal to 5°. The micro-end-milling experiment was performed to validate the existing simulation results, and the results show very good agreement.

## 1. Introduction

Machining, particularly the micro-nano fabrication technique, plays a crucial role in modern high-tech industry [1,2]. With the rapid development of micro-machine tool manufacturing techniques and cutting-edge grinding techniques, the micro-cutting process has been widely used in the fabrication of components with surface-forming accuracy in the nanoscale [3,4]. In particular, titanium and its alloys, due to their tremendous functioning properties, represent the subject of fast-growing research efforts in various fields including biomedical, instrumentation, and aerospace [5]. In order to further improve the comprehensive performance of the above parts, understanding their intrinsic mechanism during the micro-machining process has been a key challenge. On the basis of the research by Ulutan et al. [6], the typical titanium alloy micro-machined surface is composed of four parts: chip, surface white layer, subsurface plastic deformation zone, and bulk material. Recently, the states of chips and surface white layers of processed components have received much attention in academic and application fields [7,8]. However, with a demand for higher finished surface quality during the micro-cutting process, it is important to recognize that the machining dimensional accuracy and surface finish are also closely associated with the characteristics of subsurface microstructure [9]. In fact, the micro-cutting technique generates distinct process physics compared to conventional machining techniques due to the relative variable of undeformed chip thickness and tool edge radius. Specially, the above process may result in a permanent subsurface deformation layer and subsurface damages (SSDs) underlying the finished surface, which seriously impact the workpiece material mechanical properties and affect the finished surface characteristics as well as downstream technical processing [10].

On the basis of orthogonal cutting experiments on titanium alloy, many research groups have preliminarily studied the subsurface micro- and nanoscale structural features and their effects. In particular, Che-Haron et al. [11] argued that a thin subsurface damages layer was formed beneath the machined surface of titanium alloy. As tool wear increased, the depth of subsurface deformed layer increased due to the microstructural alterations. Furthermore, Ginting et al. [12] indicated that the thickness of the subsurface damages microstructure was observed to change when the cutting speed and feed rate were increased. Meanwhile, Thomas et al. [13] suggested that subsurface damages occurred in the form of dislocation slip during the micro-milling titanium alloy process. In addition, Zhang et al. [14] demonstrated the influence of various cutting depths on machined surface and subsurface topographies. Based on the X-ray diffraction detection results, they found that the micro-machining process greatly caused the change of machined surface roughness and led to subsurface microstructural evolution. Yet, despite existing experimental results that qualitatively assess certain subsurface configurations, it is difficult to quantitatively trace their formation and change laws. Therefore, computer simulation has been an incredibly effective and optimal method for examining subsurface defects layers as well as revealing their distribution and evolution. In particular, Zong et al. [15] adopted the molecular dynamics (MD) simulation method to research the transformation mechanism of workpiece microstructure for face-centered cubic crystal during the nano-cutting process. Wang et al. [16,17] was among the first to propose the large-scale MD method for characterizing workpiece subsurface deformed layers. They indicated that dislocation multiplication and motion could result in a mass of defects structure existing in the workpiece subsurface. Since the atomic-scale simulation of micro-cutting is compute-intensive, small cutting models with lengths from several nanometers to a few tens of nanometers were used to reduce the time and cost required according to previous research. Although these studies provided some scientific explanations in understanding microscale cutting, extreme machining model and twisted removal speed may result in the inaccurate conclusions.

The dislocation dynamics (DD) technique is one of the typical mesoscale simulation methods, and offers a dependable way to conduct more intricate problems within the nano- and microscale. In the DD model, a series of dislocation evolvement characteristics such as proliferation, interaction, movement, immobilization, and spatial rearrangement were tracked through constitutive rules. Although three-dimensional (3D) models were popular, a simpler two-dimensional (2D) framework has been approved as a more successful channel in a range of applications involving defect initiation and evolution behavior [18]. According to discrete dislocation dynamics theory, the authors have put forward a new trans-scale 2D cutting framework to reveal the generation mechanism of surface refinement grain in micro-machining Ti-6Al-4V [9]. Specifically, this work focuses on the influence of extrinsic tool structure parameters on the distribution and alteration of machined subsurface damages layers.

## 2. Methods

In this work, an anisotropic dislocation dynamics-based trans-scale numerical framework was explored to detect the formation mechanism of the subsurface damages layer in the processing of micromechanical structures. The model defined in this section consists of a nanoscale and a continuum scale. In the former, a kind of discrete dislocation dynamics model combining dislocation glide, climb, junction, accumulation, recovery, and annihilation was proposed to conduct a quantitative calculation of short-range interactions. In the latter case, an open-source finite element package was performed to obtain the long-range driving force imposed on individual dislocation during the micro-cutting process. 

The finite element explicit platform was used to highlight the physical comprehension of subsurface formation in the orthogonal cutting of titanium alloy. In order to obtain the external applied stress of finite element nodes in the workpiece substrate, an explicit numerical integration rule along with diagonal element mass matrices were employed with a time step of 0.5 ns. Meanwhile, a finite element reprocessing algorithm redeveloped by python scripting was used to obtain the stress distribution of element nodes quantitatively. Then, the image stress *σ^NoD^* on each of the nodes was computed in the corrector phase by subtracting intrinsic stress caused by ambient defects. Subsequently, the elastic driving load of dislocation *i* was obtained according to the interpolation algorithm. Lastly, the results in each incremental step were input periodically into the DD model, which regards workpiece material plastic deformation as the collective behavior of dislocations. In order to optimize the contact relation between the cutting tool and material matrix, a multi-component cutting model was developed, considering uncut chip thickness, tool-tip passage zone, workpiece subsurface substrate, and insert active component. Meanwhile, the reduced integration element CPE4RT was utilized for the coupling analysis between temperature and displacement. It is worth mentioning that the completely damaged elements in the damage zone are removed, while the remaining parts are identified as chips during the cutting process. The cutting speed and axial depth of cut were fixed at 10 m/s and 1 μm, respectively. The interaction mode between the cutting tool and workpiece was dry cutting. Since the material of the cutting tool was polycrystalline diamond, the insert active component was set as a rigid body. The contact conditions for the simulation are shown in Table 1. To illustrate the whole evolution process of subsurface damages structure, the cutting distance was configured as 30 μm. The acronyms employed in present work are presented in Table 2.

In comparison to conventional machining operations, the micro-processing mode always possesses a very small uncut chip thickness and depth of cut. Specifically, it is generally believed that the effective rake angle α*_t_* may be negative because the edge radius is at the same level as the uncut chip thickness, as shown in Equations (1) and (2).

**Figure 1:**
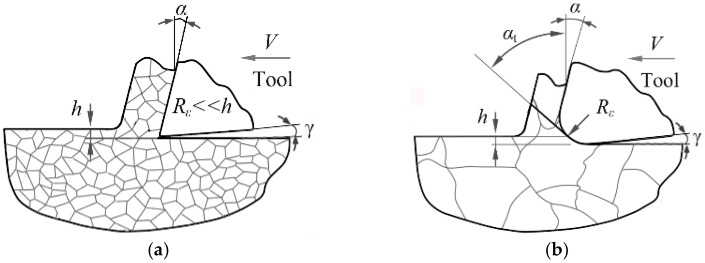
The effective rake angle during the cutting process. (**a**) Traditional machining manner; (**b**) micro-machining removal manner.

(1) αt=arcsin(h Re−1) for h<hc,

(2) $$\alpha_{t}=a\;\mathrm{for}\;h>h_{c},$$

where *R*_e_ is the tool edge radius, *h* is the uncut chip thickness, and *h*_c_ is the critical value, which can be expressed by *R*_e_ (1 + sinα). During the micro-cutting process, the cutting tool would cause high hydrostatic pressure so that the plastic extrusion deformation becomes the principal removal mechanism of the workpiece material in front of the cutting edge, as shown in Figure 1. According to plasticity theory, the vertical squeezing effect caused by the tool would induce severe subsurface microstructure alteration and hence store many SSDs in the finished surface, which was always neglected in micro-machining. Therefore, the systematic investigation of the cutting tool structure on SSD depth and distribution was further performed in the following sections. 

During the micro-cutting Ti-6Al-4V process, the workpiece in the annealed condition was adopted, and its microstructure mainly consisted of equiaxed α phase grains and a small quantity of β phase. On the basis of Wang et al. [19], the effect of the β phase can be ignored because of its small volume fraction in Ti-6Al-4V alloy. Therefore, only equiaxed α phase grains were used in the present study, and the grain size was 3.4 μm × 2.95 μm. Furthermore, the pre-existing dislocation density was 6.25 × 10^12^ m^−2^. The dislocation sources density and dislocation obstacles density were 4.2 × 10^13^ m^−2^ and 2.1 × 10^13^ m^−2^, respectively. Specifically, the workpiece subsurface damages layer was composed primarily of dislocation defects because of the low stacking fault energy [9]. By construction, slip planes {1 0 −1 0} < 1 1 −2 0 > can be combined to generate an effective in-plane slip system and the reference slip directions were {0°, 60°, 120°} on the foundation of the *x*-axis [20]. Special constitutive equations were established to incorporate 3D dislocation mechanisms, such as dislocation cross-slip and junction formation, within 2D plane simulation. The above rules may be referred to as 2.5D laws [21]. In dynamics simulation, the evolution of discrete dislocation *i* was determined by Peach-Koehler (P-K) force, *f^i^*, which depends on the boundary solution for image stress field σ, as shown in Equation (3):

(3) $$f^{i}=m^{i}\cdot \left(\hat{\sigma }+\underset{j\neq i}{\sum }\sigma^{j}\right)\cdot b^{i},$$

where {σ*^i^*, *j* ≠ *i*} is the infinite medium stress fields, which can be computed on the base of transient dislocation structure. Moreover, *m^i^* is the slip plane normal and *b^i^* is the Burgers vector of dislocation *i*. 

In the nanoscale part, a point source was adopted to mimics the Frank–Read (F-R) initiation mechanism from pinned segments on out-of-plane slip planes. Many dislocation sources were randomly distributed in the slip systems with density-specific resources *ρ*_nuc_. A discrete F-R source can multiply a dipole of dislocations when the Peach-Koehler force exceeds intrinsic nucleation strength during a period of nucleation time. In particular, in the present 2.5D framework, the initiation time and threshold of screw segment were both larger than that for edge dislocation [22]. In addition, a random distribution of dislocation obstacles was contained in the workpiece substrate, which symbolized either many impurity precipitates on slip planes or junction dislocations on out-of-plane glide systems. Particularly, as soon as the climb distance exceeded the module of the Burgers vector, the piled-up dislocation was permitted to move outside the initial glide plane [23]. Dislocations with opposite signs would be annihilated if they came into the specific distance of 6***b***.

## 3. Simulation Results and Discussion

### 3.1. Influence of Cutting Distance on Subsurface Microstructure

**Figure 2:**
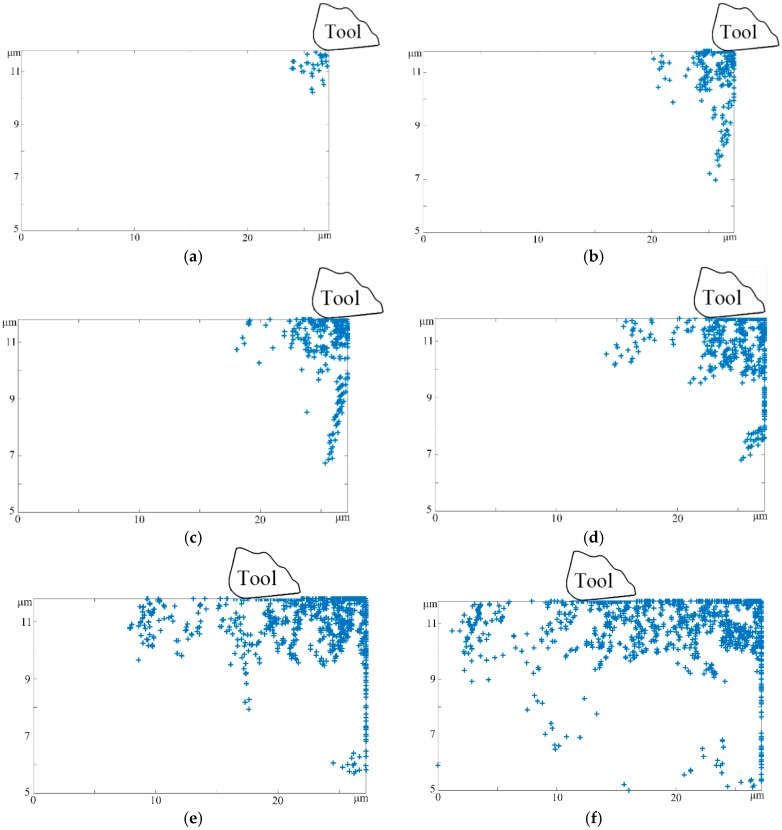
The subsurface defects microstructural evolution with different cut distances (cutting speed 10 m/s, cutting depth 1 μm, tool edge radius 0.25 μm, rake angle 5°, and clearance angle 15°). (**a**) cutting distance 0.5 μm; (**b**) cutting distance 1.5 μm; (**c**) cutting distance 2.5 μm; (**d**) cutting distance 5 μm; (**e**) cutting distance 10 μm; (**f**) cutting distance 15 μm.

During the micro-cutting process, the process of material removal and finished surface formation results in plenty of linear defects and residual stress in the subsurface plastic deformation zone, which eventually leads to the formation of a damages layer. Therefore, in order to further investigate the complex dislocation defects nucleation, evolution, accumulation, and annihilation taking place in the subsurface area, a series of subsurface dislocation dynamics snapshots were specially extracted to indicate the variation tendency of surface and subsurface damage distribution, as shown in Figure 2.

In the inception phase, we consider that some discrete dislocations were generated in the support zone of the workpiece after the tool cuts in the Ti-6Al-4V substrate, as illustrated in Figure 2a. Meanwhile, the instantaneous impulse load and thermal stress within titanium alloy crystal caused small amounts of pre-existing dislocations to be unable to accommodate the external effect in a timely manner. As a result, the discrete F-R dislocation sources in the immediate subsurface of the workpiece have to emit continuous dislocation dipoles, as shown in Figure 2b. Furthermore, the extrusion action of the cutting tool would form a powerful hydrostatic pressure field, which produces a transient cutting force wave into the bulk material in the orthogonal cutting process of titanium alloy [24]. This results in the formation of many dislocation lines took shape in the deep-surface zone, as illustrated in Figure 2c, as these lines were along the [1 1 −2 0] and [−1 −1 2 0] directions. Specifically, from the subsurface defect structure detail information, the coordinate values of the dislocations on the top layer and the bottom layer can be obtained. The difference of two coordinate values was regarded as the thickness of the subsurface damages layer, which was used as an index to evaluate the subsurface microstructure. From Figure 1, it can be seen that the depth of the defect structure increased significantly at the beginning. Then it remained in a stable, high level because the dislocation nucleation and annihilation caused both ends to meet at the stable stage of the micro-cutting process. 

In comparison to the traditional removal technique, the microscale effect not only produced more complicated thermos-mechanical effects, but also resulted in a larger interaction region during the micro-cutting process. This causes a large amount of macro residual stress to be stored in the machined surface and subsurface, which further results in subsequent dislocation proliferation. Similarly, the workpiece subsurface damage structure could generate micro inner stress, which leads to the increase of dislocations. Therefore, we can conclude that the finished subsurface remained a mass of dislocations, even if the tool tip was removed.

As mentioned above, the depth of the subsurface damages layer would eventually reach a plateau when the removal distance was larger 5 μm. In this stage, it is worth mentioning that the subsurface plastic deformation was composed of dislocation proliferation in the near-surface deformation zone as well as the dislocation slip and increase in the deep-surface deformation zone, as shown in Figure 2d. The forward movement of the cutting tool and the complex dislocation movement caused massive atomic migration, which finally led to the generation of a rough processed surface. From Figure 2e, screw and edge dislocations were generally weaving and intertwining with the increase of the cutting length, which can induce cross-slip dislocations and dislocation locks phenomena. In particular, the above extrinsic barriers and inherent crystal impurities hindered the subsequent dislocation evolution and formed a subsurface work-hardening layer. In addition, due to the subsurface atomic motion and removal, many vacancy defects were gathered in the position of dislocation migration and annihilation, respectively. The synthetic effect of vacancy energy and cutting energy ultimately caused dislocation glide bands and refinement grains, as shown in Figure 2f. As the residual stress would change continuously during and after the passing of the tool, the dislocation density would change gradually. After cutting, the subsurface defects would partially recover back or move out to the machined surface, which resulted in the decrease of the density of dislocations in the lower right region [25,26].

### 3.2. Influence of Tool Edge Radius on Subsurface Microstructure

**Figure 3:**
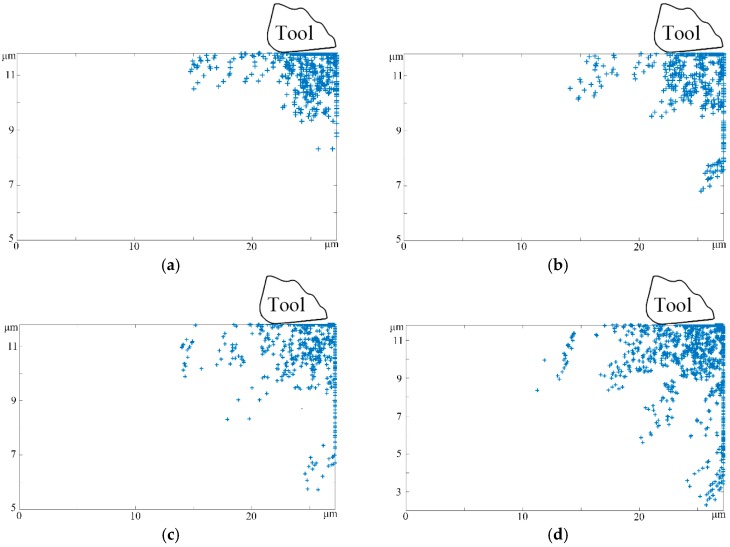
The subsurface defects microstructural evolution with different tool edge radii (cutting distance 5 μm, cutting speed 10 m/s, cutting depth 1 μm, rake angle 5°, and clearance angle 15°). (**a**) tool edge radius 0 μm; (**b**) tool edge radius 0.25 μm; (**c**) tool edge radius 0.5 μm; (**d**) tool edge radius 1 μm.

Typical morphologies of subsurface damages layers produced with various tools in the micro-cutting process were arrayed with respect to different edge radii. When the edge radius was far less than the depth of cut, as depicted in Figure 3a, very few dislocations were multiplied and distributed in the rear of the near-surface shearing zone, which can be regarded as a characteristic feature of the subsurface microstructure during the conventional cutting process. However, once the independent cutting depth moved closer to the critical value of the micro-machining mode, it can be seen that increasing the tool edge radius would increase the number of SSDs simultaneously. In addition, as illustrated in Figure 3b–d, although the subsurface damages layers were generated under various tool parameters, all of them consisted of two different appearances: intertwining dislocation bands and parallel dislocation lines. 

Since the effective rake angle always assumes a large negative value in the processing of surface micromechanical structures, the local workpiece matrix material ahead of the cutting tool was downwardly suppressed. Specifically, the compressive stress component is equally dominant, which provides a stress field similar to the hydrostatic stress state. With the increment of the cutting edge radius, the distribution of high stress areas changed correspondingly. Hence, the effect of thrust force gradually increased and the subsurface microstructure distortion became severer. Furthermore, as the tool edge radius grew and finally was equal to the value of the uncut chip thickness, we considered that the subsurface damages layer formed apparent persistent slip bands (PSBs) in the anterior regions of the cutting edge, which initially interconnected with each other and then became isolated as the cutting length increased, as in Figure 3d. It is noteworthy that the crowding in or out effect among PSBs may cause the extreme non-uniform phenomenon of workpiece material strain distribution, which may result in the initiation of subsurface microcracks.

According to the measurement of the subsurface damages layer, the evolution law of the subsurface with the changes in the tool edge radius was obtained. The thickness of the subsurface damages layer was thin and stable when the tool edge radius was 0 μm. Nevertheless, as the edge radius increased, the energy dissipation induced by local temperature rising made a serious impact on the subsurface substrate, which not only enhanced the nucleation rate of dislocation sources, but also boosted the self-diffusion constant of dislocation and then improved its liberating ability from pinning obstacles. Therefore, the depth of the subsurface damages layer increased significantly until reaching the bottom of the workpiece matrix, as shown in Figure 3d.

### 3.3. Influence of Tool Rake Angle on Subsurface Microstructure

**Figure 4:**
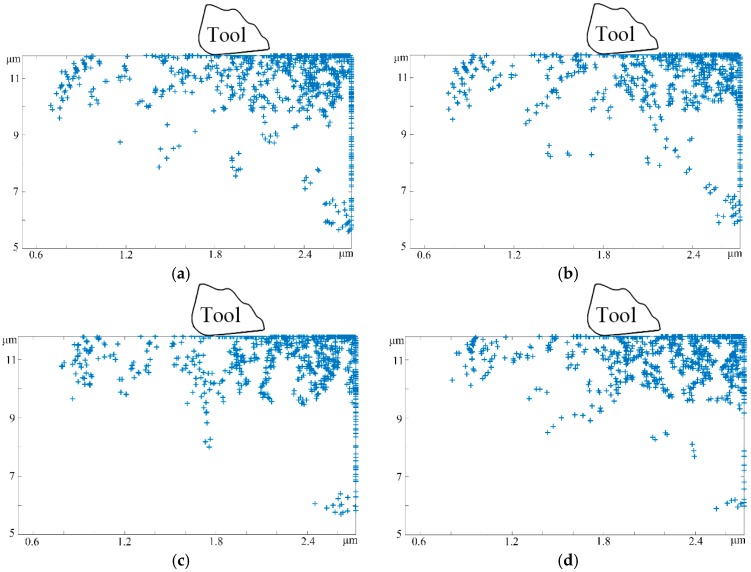
The subsurface defects microstructural evolution with different rake angles (cutting distance 10 μm, cutting speed 10 m/s, cutting depth 1 μm, tool edge radius 0.25 μm, and clearance angle 15°). (**a**) rake angle 1°; (**b**) rake angle 3°; (**c**) rake angle 5°; (**d**) rake angle 10°.

On the basis of various tool structural parameter classifications, tool rake angles of 1°, 3°, 5°, and 10° were adopted to study machined surface and subsurface microstructure changes, while the other processing parameters were kept the same, such as the cutting distance of 10 μm, cutting speed of 10 m/s, and cutting depth of 1 μm. During the micro-cutting process, the influence of the tool rake angle α on the workpiece material lies primarily in two areas. First, as previously mentioned, it can be adopted to determine the value of the effective rake angle α*_t_*. On the other hand, we considered that the tool rake angle plays a critical part in the generation of chips. In the micro-machining process, the shear angle continually changes with the evolution of the rake angle. Increasing the rake angle would greatly weaken the shearing effect between the tool rake face and uncut material, which provides an effective channel to restrain both chip deformation and friction action in the vicinity of the cutting edge, and eventually leads to the loss of coverage of principal stress and thrust stress. Therefore, the amount and type of dislocations in the subsurface damages layer both decreased significantly as the rake angle increased in the middle stage of the machining process, as shown in Figure 4a–d.

Moreover, it can be seen that the changes in the tool rake angle have great impacts on the workpiece subsurface microstructure at the moment that cutting tool makes a cut. Hence, by comparing Figure 4a,d, it can be seen that the tool of 1° would generate more dislocation defects and lines than the tool of 10° in the deep-surface substrate. Furthermore, the lower rake angle at the same cutting distance results in a larger region of heat dissipation. The underlying reason for this is that both the chip deformation and cutting energy consumption decreases with the increment of the tool rake angle, which narrows down the range of the cutting temperature at the same time. Not only that; the heat dissipation severely affects the workpiece subsurface damages layer, which further results in thermal activation-induced dislocation nucleation and increases the probability of subsurface damages, leaving dislocation bands and grain boundaries dominating the subsurface damages layer morphology.

### 3.4. Influence of Clearance Angle on Subsurface Microstructure

**Figure 5:**
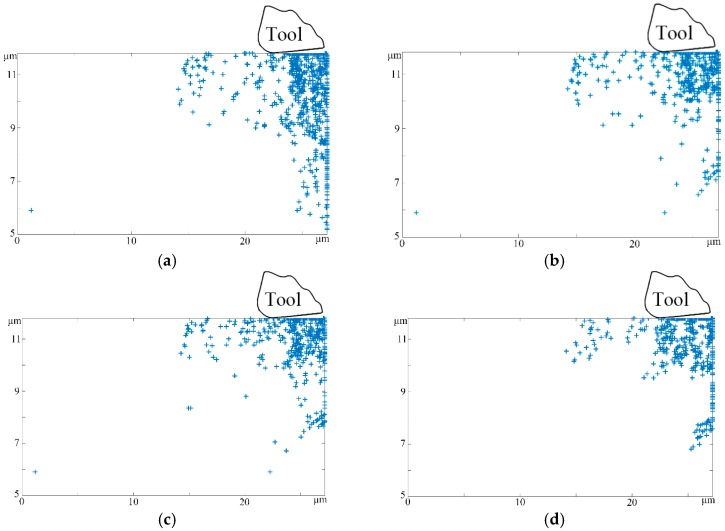
The subsurface defects microstructural evolution with different clearance angles (cutting distance 5 μm, cutting speed 10 m/s, cutting depth 1 μm, tool edge radius 0.25 μm, and rake angle 5°). (**a**) clearance angle 0°; (**b**) clearance angle 5°; (**c**) clearance angle 8°; (**d**) clearance angle 15°.

Figure 5 presents the instantaneous structures of subsurface deformed with different clearance angles when the removal length is 2.5 μm. It is known that the workpiece surface was in a complicated stress field subjected to the interaction of compressive load and shear load. Actually, the high compression stress also existed in the subsurface damages layer, which is derived from the flank contact zone. Specially, as the cutting tip passed through workpiece, the variety of the tool clearance angle is vital for the subsequent recovery process of the finished surface and subsurface microstructure. From Figure 5, we can see that the type and quantity of subsurface defects both decreased sharply with the increment of the clearance angle. This can be explained by the fact that the horizontal clearance angle of the cutting tool would bring secondary hydrostatic pressure into the machined surface, which further transforms the subsurface damages structural distribution and activates residual F-R dislocation sources. In the meantime, the nucleating dislocations beneath the flank surface would result in sufficient extension, movement, accumulation, and annihilation if the clearance angle is small enough, as shown in Figure 5a. As a result, an array of residual defects would remain in the subsurface damages layer. In addition, a smaller clearance angle usually occurred with a larger friction region between the flank surface and material surface, thus leading to the increase of the processed surface temperature. These factors further decreased the nucleation strength of subsurface dislocation and improved the dislocations’ climb ability. Therefore, it is easy to see that the subsurface substrate in Figure 5a has a far greater quantity and density of defects compared to others.

It is noteworthy that, although increasing tool clearance angle significantly reduced the thickness of the subsurface damages layer, it remained fairly static when the clearance angle was equal to or greater than 5°. Specifically, the fresh workpiece surface formed by squeezing would not bear any ironing effect if too large of a clearance angle was found, which may promote rough machined surfaces. As a result, considering the influences of clearance angle on the machined surface finish and subsurface damages layer structural evolution, good structure parameters would be obtained while maintaining the processing quality.

## 4. Experimental Results

**Figure 6:**
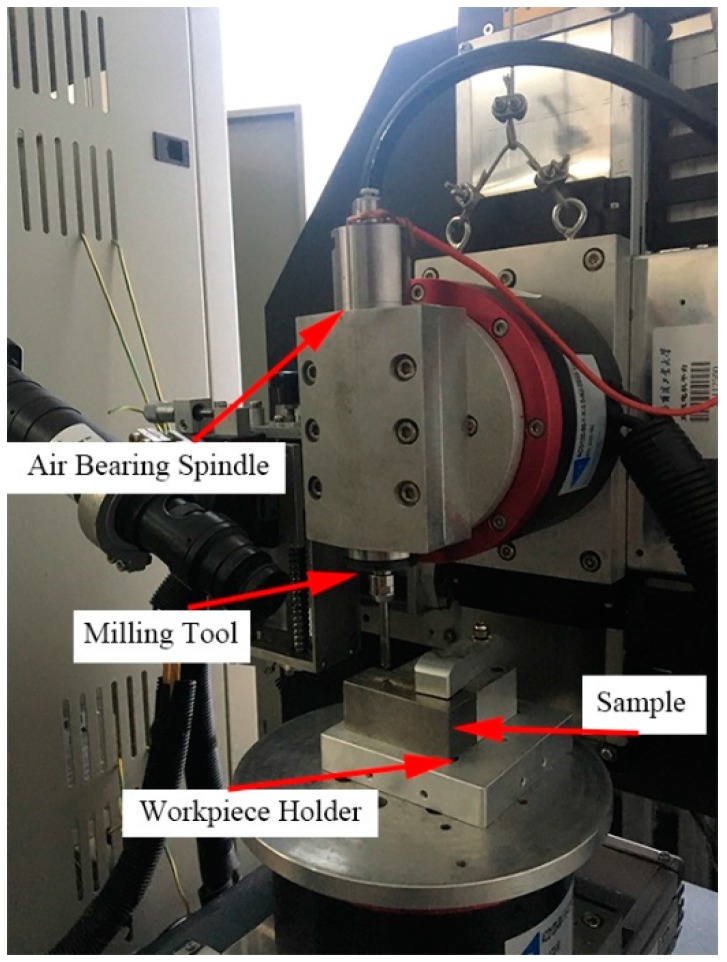
The experimental facility for milling titanium alloy samples.

**Figure 7:**
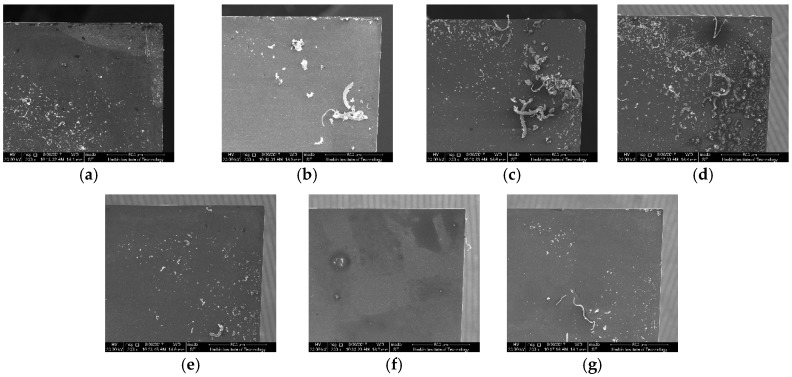
The morphology of mills obtained by Scanning Electron Microscopy (SEM). (**a**) Test No. 1; (**b**) Test No. 2; (**c**) Test No. 3; (**d**) Test No. 4; (**e**) Test No. 5; (**f**) Test No. 6; (**g**) Test No. 7.

The micro-end-milling experiments were performed on a five-axis milling numerical control machine tool, as shown in Figure 6. The overall dimensions of the micro-machine tool were 580 mm × 500 mm × 770 mm. Meanwhile, the machine tool was equipped with three precision linear axes and two rotational axes, which were driven by servo motors with a maximum speed of an air-bearing spindle (16 × 10^4^ rpm) and a positional precision of ±0. 35 μm/10 mm. A titanium alloy (Ti6Al4V) sample was clamped on a ground metal plate, the dimensions of which were 40 mm × 20 mm × 20 mm. Identical tool structural parameters of brand polycrystalline diamond (PCD) micro-end mills were used in both the multi-scale simulation and machining experiments in Table 3. The morphology of the mills obtained by Scanning Electron Microscopy (SEM) is presented in Figure 7. The machining parameters were: depth of cut of 15 μm, spindle speed of 25,000 rpm, and feed per tooth of 1 μm. After cutting, the slot surfaces were measured by a white light interferometer (Talysurf CCI 2000, Taylor Hobson, Leicester, UK).

**Figure 8:**
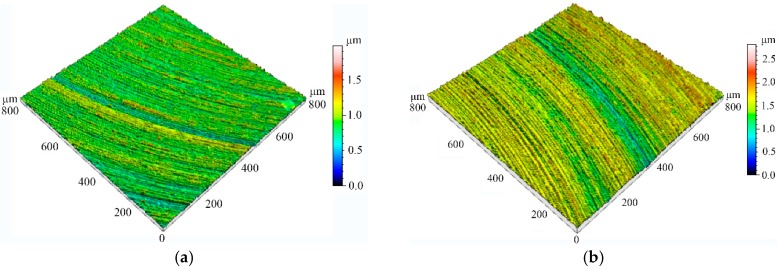
Selected machined surface local detail topography. (**a**) Test No. 1; (**b**) Test No. 3.

To further reveal the influence of tool structural parameters on the surface finish, selected measured finished topography features are shown in Figure 8. It can be noted that the variation of tool design parameters would significantly affect the overall performance of machined slots. Specifically, the measured surface roughness indicated that increasing the cutting edge radius would produce tremendous negative impacts on the machined surface finish. Furthermore, the surface roughness was apt to deteriorate with the decrease of the tool rake angle. When the rake angle changed from 5° to 1°, there was around a 47.5% increase in the surface finish value. As expected, increasing tool clearance angle had little impact on the finished surface quality if it was greater than or equal to a certain vale. In conclusion, a good agreement between the measured surface topography and simulation results was obtained.

## 5. Conclusions

In the present work, a 2.5D climb-assisted dislocation dynamics-based model was designed to obtain detailed insight into the subsurface damages structure evolution during the micro-cutting process of titanium alloy. The present study has a focus on the dependency between tool structure parameters and subsurface defects. The novel results can be summarized as follows:

(a) In the process of micro-cutting, the dislocation multiplication, activity, pile-up, and annihilation resulted in many defects existing in the subsurface of the workpiece, such as discrete edge and screw dislocations, double cross-slip dislocations, parallel dislocation lines, intersection slip bands, vacancy defects, and refinement grains.
(b) A reliable measurement method of the thickness of the subsurface damages layer was put forward during the micro-machining process. When the tool intrinsic parameters and processing conditions were kept constant, the depth of the subsurface damages layer increased significantly in the initial stage. Consequently, it would be maintained at a stable high-level because the dislocation nucleation and annihilation would make both ends meet in the stable removal phase.
(c) Upon increasing the tool radius, the number of subsurface defects sharply increases beneath the cutting edge. Instead, the larger radius causes serious impact load and energy dissipation into the workpiece, which results in PSBs and the thermal activation dislocation of the core in the subsurface matrix material.
(d) Increasing the tool rake angle greatly weakens the squeezing action between the tool and workpiece substrate, which eventually inhibits the energy consumption. In addition, although increasing the tool clearance angle significantly decreases the depth of the subsurface damages layer, it almost maintains a stable value if the clearance angle is greater than or equal to 5° in the cutting process of Ti-6Al-4V titanium alloy. Furthermore, the comparison between the predicted results and the experiment showed very good agreement.

## Figures and Tables

**Table 1:** Contact conditions for the simulation.

Contact Parameters	Value
Heat transfer coefficient (W/m^2^·K)	4 × 10^4^
Heat partition coefficient	0.5
Friction coefficient	0.7
Friction energy transferred into heat	100%

**Table 2:** Acronyms involved in this study.

Acronyms	Description
DD	Dislocation dynamics
SSDs	Subsurface damages
MD	Molecular dynamics
FE	Finite element
F-R	Frank–Read
P-K	Peach-Koehler
PCD	Polycrystalline diamond

**Table 3:** Experimental matrix of micro-machining of titanium alloy and responses: measured surface roughness.

Test No.	Tool Diameter (mm)	Cutting Edge Radius (μm)	Rake Angle (°)	Clearance Angle (°)	Surface Roughness (nm)
1	4.5	20	5	15	70.6
2	4.5	30	5	15	103.9
3	4.5	40	5	15	121.9
4	4.5	20	3	15	88.7
5	4.5	20	1	15	104.2
6	4.5	20	5	8	77.8
7	4.5	20	5	5	93.3
